# Supplementary material for: Blood Clotting Dissolution in the Presence of a Magnetic Field and Preliminary Study with MG63 Osteoblast-like Cells—Further Developments for Guided Bone Regeneration?
Source: Bioengineering (Basel). 2023 Jul 26;10(8):888. doi: 10.3390/bioengineering10080888 (PMC10451701; doi:10.3390/bioengineering10080888)
Supplement: Supplementary file 1 [file bioengineering-10-00888-s001.zip › bioengineering-2445848-supplementary.pdf]

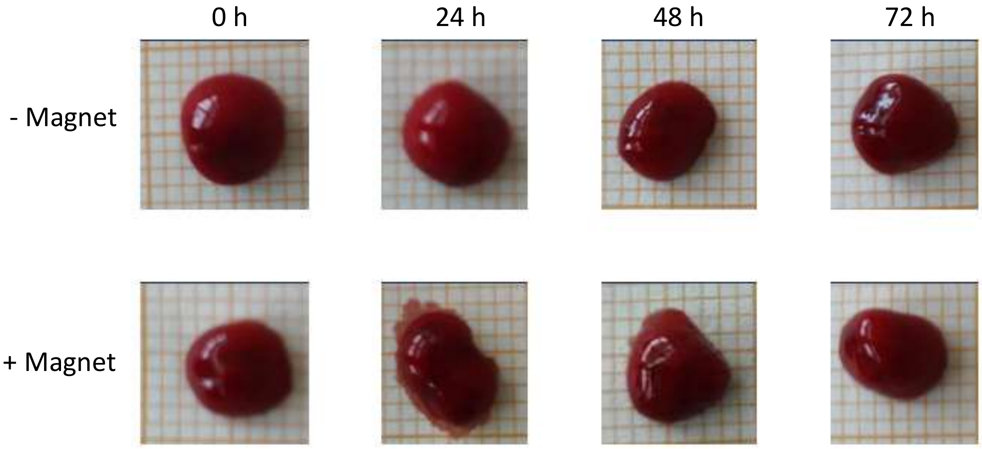

Supplemental Figure 1. Representative images of WB clots at 0h, 24h, 48h and 72h in the absence or presence of the magnetic field.
